# The clinical value of multimodal neuroimaging in monoclonal antibody therapy for Alzheimer's disease

**DOI:** 10.3389/fneur.2026.1761380

**Published:** 2026-05-08

**Authors:** Yingte Wang, Hong Li, Jing Zhou, Saiyao Zhao, Airong Yang, Zhiming Li

**Affiliations:** 1 Department of Radiology, The Second Affiliated Hospital, Guangzhou Medical University, Guangzhou, Guangdong, China; 2 Qinghai Cardio-Cerebrovascular Specialty Hospital, Qinghai High Altitude Medical Research Institute, Xining, Qinghai, China

**Keywords:** Alzheimer's disease, amyloid-related imaging abnormalities (ARIA), Monoclonal antibody therapy, multimodal neuroimaging, positron emission tomography (PET)

## Abstract

Multimodal neuroimaging plays an indispensable role in the diagnosis, monitoring, and therapeutic evaluation of monoclonal antibody (mAb) therapies for Alzheimer's disease (AD). Structural MRI (sMRI) enables early detection of cerebral atrophy and amyloid-related imaging abnormalities (ARIA), a critical adverse effect associated with anti-Aβ immunotherapies. Positron emission tomography (PET) provides direct visualization and quantification of Aβ plaque clearance, serving as an objective biomarker of target engagement. Functional MRI (fMRI) has been investigated as a means to detect dynamic changes in brain network connectivity following treatment, though the evidence remains preliminary. The integration of these modalities significantly enhances diagnostic accuracy and allows for personalized assessment of treatment response. Furthermore, artificial intelligence (AI) technologies improve the efficiency and predictive power of imaging data analysis, supporting clinical decision-making. Despite these advances, challenges remain regarding the sensitivity and specificity of current imaging techniques, heterogeneity in treatment responses, and the need for long-term safety monitoring. Standardized imaging protocols, combined with multidisciplinary collaboration and robust AI-assisted modeling, are essential to optimize therapeutic outcomes and minimize risks in mAb-based AD treatment.

## 1 Introduction

Alzheimer's disease (AD) represents the most prevalent form of neurodegenerative disorder and dementia, with its prevalence demonstrating a concerning upward trajectory. It is projected that the global number of individuals living with AD will reach a staggering 150 million by 2050 (1). Beyond imposing severe cognitive impairments on patients, AD poses substantial socioeconomic burdens, accounting for approximately 1% of the global economic output (2). The disease is characterized by two hallmark neuropathological features: aberrant deposition of β-amyloid (Aβ) plaques and hyperphosphorylation of tau proteins, which collectively drive neuronal injury and progressive cognitive decline (3).

Monoclonal antibodies (mAbs) targeting β-amyloid have ushered in a breakthrough in AD therapeutics, representing the first class of disease-modifying therapies capable of slowing clinical decline through intervention in the core biological processes of the disease (4). To date, anti-Aβ monoclonal antibodies including lecanemab and donanemab have received traditional approval from the US Food and Drug Administration (FDA) for the treatment of early Alzheimer's disease, whereas aducanumab was approved via the accelerated approval pathway but is no longer commercially marketed (5). These antibodies significantly reduce cerebral amyloid plaque burden by selectively engaging with various forms of Aβ aggregates (6, 7). Clinical studies demonstrate that anti-Aβ monoclonal antibodies partially reverse amyloid pathology and may alter disease progression by decelerating or halting clinical decline (7, 8). However, these treatments demonstrate limited efficacy in improving cognitive function, with a substantial proportion of patients experiencing adverse effects such as amyloid-related imaging abnormalities (ARIA) (9, 10). Amyloid-related imaging abnormalities (ARIA) are classified into two main types: ARIA-E (edema or effusion) and ARIA-H (hemosiderin deposition, including microhemorrhages and superficial siderosis).

**Figure 1:**
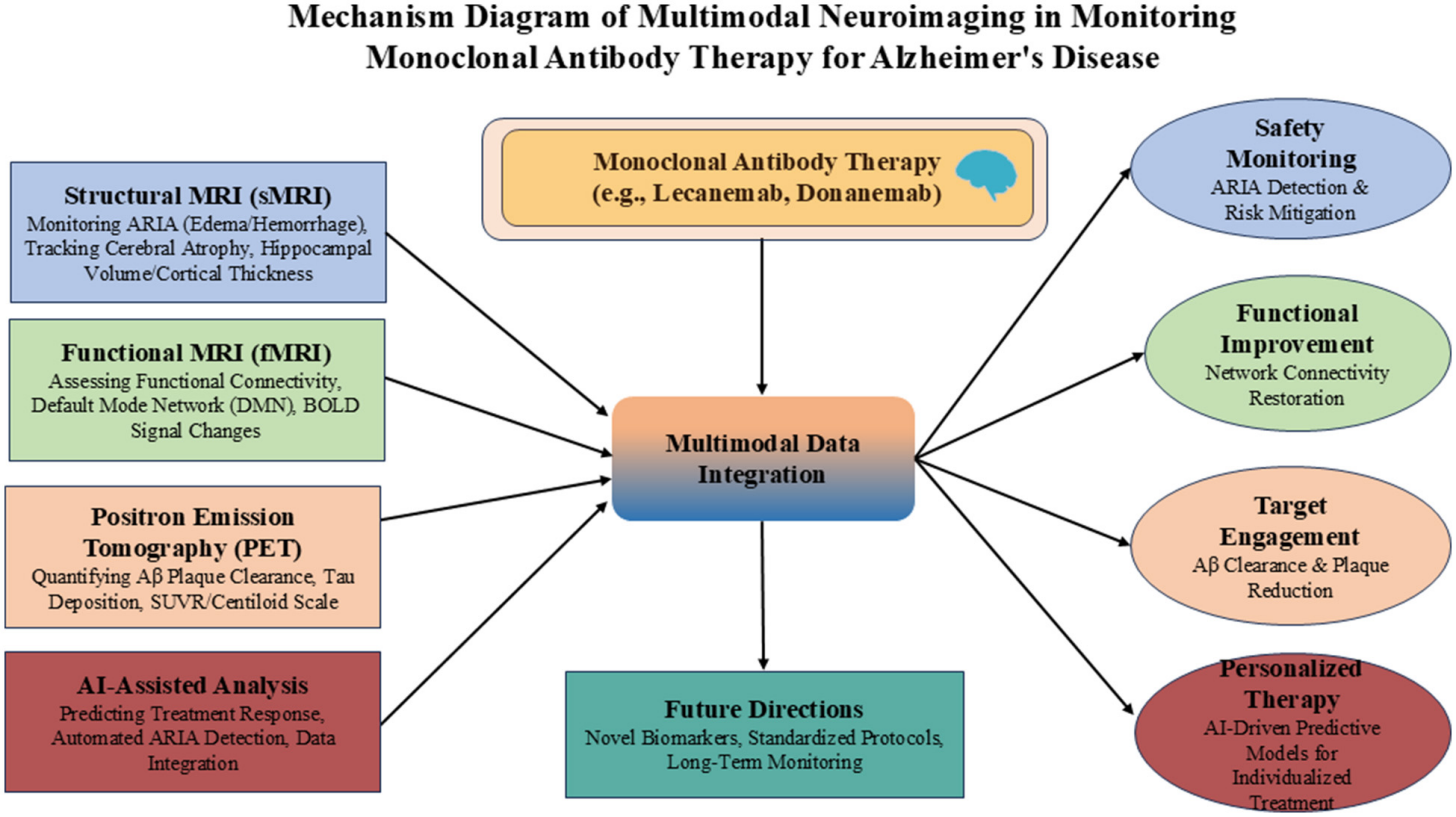
Comprehensive framework of multimodal neuroimaging in monoclonal antibody therapy for Alzheimer's disease.

Multimodal neuroimaging techniques are playing an increasingly pivotal role in the diagnosis and management of Alzheimer's disease. Imaging modalities such as magnetic resonance imaging (MRI) and positron emission tomography (PET) enable the detection of subtle alterations in brain structure and function, providing critical evidence for AD diagnosis (11, 12). In addition, anti-Aβ monoclonal antibody therapies require regular cerebral MRI monitoring to detect treatment-emergent adverse events such as amyloid-related imaging abnormalities (ARIA) (13, 14). Multimodal imaging fusion approaches that integrate complementary biomarkers from MRI, PET, and cerebrospinal fluid assays significantly enhance diagnostic performance for AD (15). Nevertheless, current neuroimaging techniques face persistent challenges including insufficient sensitivity and limited specificity, while the high cost of PET examinations frequently results in missing data. Furthermore, the effective integration of multimodal imaging data to guide personalized therapeutic decisions remains an unresolved critical issue (16, 17). The integrated role of multimodal neuroimaging across diagnosis, therapeutic monitoring, and personalized assessment is summarized in Figure 1.

## 2 Core molecular and mechanistic research advances

### 2.1 Pathological roles of β-amyloid and tau proteins in Alzheimer's disease

The defining neuropathological hallmarks of Alzheimer's disease (AD) comprise the cerebral deposition of β-amyloid (Aβ) plaques and the formation of neurofibrillary tangles composed of hyperphosphorylated tau protein (18). Preclinical and clinical evidence indicates that the interplay between Aβ and tau pathologies drives the entire continuum of AD pathological progression. As a key initiating factor in AD, the neurotoxic mechanisms of Aβ remain incompletely elucidated through existing methodologies. A complex network of interactions exists between Aβ and tau abnormalities during AD pathogenesis, with the immune system appearing to be profoundly involved in both pathological processes (17). These interactions may be centrally mediated by the apolipoprotein E ε4 allele, which serves as a critical molecular link connecting Aβ accumulation to tau hyperphosphorylation (17). Genetic studies further confirm that plasma Aβ species (including amyloid precursor protein, amyloid-like protein 2, serum amyloid P component, and Aβ peptides) alongside cerebrospinal fluid levels of Aβ, total tau, and phosphorylated tau exhibit significant associations with AD risk (18).

### 2.2 Mechanisms of action of lecanemab and donanemab

Monoclonal antibodies targeting β-amyloid (Aβ), including lecanemab and donanemab, represent a significant breakthrough in the therapeutic landscape for Alzheimer's disease (19). These antibodies exert their effects through specific targeting of distinct Aβ species. Lecanemab (BAN2401) demonstrates selective clearance of Aβ protofibrils (6), while long-term cerebral distribution studies of RmAb158 (the murine recombinant version of lecanemab) reveal effective blood-brain barrier penetration and sustained intracranial persistence, which may constitute a key mechanism underlying its therapeutic efficacy (20). Donanemab, on the other hand, is a monoclonal antibody that specifically targets N-terminal truncated pyroglutamate-modified Aβ (N3pG-Aβ), a form enriched in mature amyloid plaques. By binding to these plaque-core epitopes, donanemab promotes microglial-mediated clearance of existing fibrillar amyloid deposits, contributing to its pronounced plaque-reducing effect observed in clinical trials (5, 19). Similar to other anti-Aβ antibodies, donanemab requires regular cerebral MRI monitoring for treatment-emergent amyloid-related imaging abnormalities (ARIA) (5, 14). Notably, bispecific brain-penetrating antibodies can achieve sufficient intracerebral concentrations to serve dual functions as both therapeutic agents and PET tracers for diagnostic purposes (21). The US FDA approval of monoclonal antibodies targeting specific Aβ forms for AD treatment marks a crucial milestone in translating disease-modifying therapies into clinical practice, although the commercial availability of individual agents may vary based on regulatory and marketing decisions (22).

### 2.3 Impact of monoclonal antibody therapies on AD pathophysiology

Anti-Aβ monoclonal antibody therapies demonstrate dual mechanistic effects, reducing not only cerebral Aβ burden but also tau protein levels, as confirmed in both animal models and clinical trials (23, 24). Clinical studies indicate that anti-Aβ monoclonal antibodies significantly slow clinical disease progression in AD patients, with this therapeutic benefit persisting throughout 18-month observation periods (25). PET imaging assessments reveal that these treatments effectively clear cerebral Aβ deposits while improving multiple biomarker profiles, including cerebrospinal fluid Aβ42/40 ratios (26). In pivotal clinical trials, anti-Aβ monoclonal antibodies have demonstrated substantial plaque clearance. For instance, lecanemab treatment resulted in a mean reduction of the standardized uptake value ratio (SUVR) for cerebral amyloid burden by 59.1% (95% CI: 56.3 to −61.9%) from baseline at 18 months as measured by Aβ-PET, with a clear dose-dependent effect (6). In the donanemab phase 3 trial, the mean reduction in amyloid plaque burden (measured by centiloid scale) was 85.1% (95% CI: 83.4 to −86.8%) from baseline at 18 months, and the proportion of patients achieving amyloid PET-negative status (defined as a centiloid level <24.8) was approximately 60% at 12 months (5). Cognitive benefits, however, were more modest: lecanemab treatment yielded a standardized effect size of −0.45 (95% CI: −0.67 to −0.23) on the Clinical Dementia Rating–Sum of Boxes (CDR-SB), corresponding to an approximate 27% slowing of clinical decline (4). The incidence of amyloid-related imaging abnormalities (ARIA) exhibited dose- and APOE ε4-dependent patterns. In the lecanemab phase 3 trials, ARIA-E (edema) occurred in 21.5% (95% CI: 18.2 to −25.2%) of APOE ε4 carriers vs. 9.2% (95% CI: 7.1 to −11.8%) of non-carriers; ARIA-H (microhemorrhages) were observed in 17.5% (95% CI: 14.5 to −21.0%) and 7.5% (95% CI: 5.6 to −9.9%), respectively (14). These data underscore the heterogeneity of treatment responses across genetic backgrounds and disease stages, highlighting the need for individualized therapeutic strategies. However, despite substantial reduction in Aβ burden achieved with anti-Aβ antibody therapies, their effects on cognitive improvement remain limited (27, 60). Furthermore, a considerable proportion of patients receiving Aβ-targeted antibodies experience adverse events such as amyloid-related imaging abnormalities (ARIA). Emerging research indicates that ventral tegmental area (VTA) electrical stimulation reduces accumulation of phosphorylated tau and Aβ in AD model animals, while concurrently decreasing oxidative stress and inflammatory markers, thereby providing novel insights for developing combined therapeutic strategies.

## 3 Application of multimodal neuroimaging in AD diagnosis

### 3.1 Structural MRI (sMRI) in early AD diagnosis

Structural MRI (sMRI) has emerged as a critical tool for the early diagnosis of Alzheimer's disease, enabling precise detection of cerebral atrophy patterns. sMRI quantifies characteristic structural alterations in AD, such as hippocampal volume reduction and entorhinal cortex thinning, which often precede clinical symptoms (28). The specific atrophy in these regions serves not only as a core imaging biomarker for AD but also shows a trajectory closely linked to the rate of cognitive decline, providing a basis for objective disease staging (12, 18). Studies demonstrate that sMRI-detected structural changes correlate significantly with cognitive decline, providing objective evidence for disease staging. Furthermore, sMRI plays a unique role in monitoring monoclonal antibody therapies by detecting amyloid-related imaging abnormalities (ARIA), a significant adverse effect associated with anti-Aβ antibody treatment (13, 14). Beyond monitoring ARIA, sMRI is crucial for assessing treatment response. It can track longitudinal changes in brain atrophy, although short-term volume reductions must be interpreted with caution as they may reflect “pseudo-atrophy” due to amyloid plaque clearance rather than actual neuronal loss.

### 3.2 Functional MRI (fMRI) for assessing functional connectivity

Functional MRI (fMRI) non-invasively evaluates alterations in brain functional connectivity in AD patients through blood-oxygen-level-dependent (BOLD) signal detection. fMRI studies reveal disruptions in default mode network connectivity, detectable even in early disease stages (29). This connectivity impairment is not limited to the DMN but gradually affects frontoparietal networks governing executive function and attention, with specific abnormal patterns correlating with impairments in distinct cognitive domains (17, 26). As the disease progresses, fMRI captures neural network reorganization and compensatory activation patterns, offering valuable insights into disease mechanisms. Resting-state fMRI is particularly valuable for detecting subtle functional changes that may precede measurable structural or cognitive decline. In therapeutic monitoring, fMRI has been explored to assess functional network changes following monoclonal antibody interventions, with preliminary studies suggesting potential as a functional correlate of treatment response (25, 27); however, robust and reproducible evidence for fMRI-based biomarkers in anti-amyloid trials remains limited. Furthermore, advanced analytic approaches like graph theory applied to fMRI data can quantify the efficiency and resilience of brain networks, offering a more nuanced understanding of treatment-induced functional restoration.

### 3.3 PET imaging for detecting Aβ and tau deposition

Positron emission tomography (PET) remains the gold standard for detecting AD pathological hallmarks, including Aβ plaques and tau neurofibrillary tangles. Aβ-PET imaging visually maps cerebral amyloid distribution, informing patient selection for monoclonal antibody therapies (30). Tau-PET evaluates neurofibrillary tangle burden, with abnormal deposition patterns closely correlating with cognitive impairment severity (31). PET imaging is pivotal in treatment monitoring, objectively assessing amyloid clearance post-therapy—e.g., lecanemab treatment significantly reduces Aβ-PET signal after 18 months (32). PET biomarkers also serve as surrogate endpoints in clinical trials, accelerating drug development. Fluorodeoxyglucose positron emission tomography (FDG-PET) provides a surrogate measure of synaptic activity and neuronal function by quantifying regional cerebral glucose metabolism. In Alzheimer's disease, FDG-PET typically reveals a characteristic pattern of hypometabolism in the temporoparietal cortex, posterior cingulate, and precuneus, which often precedes structural atrophy and correlates with cognitive impairment severity (29). Unlike Aβ-PET and tau-PET, which directly detect pathological protein aggregates, FDG-PET captures downstream functional consequences of neurodegeneration, offering complementary information for differential diagnosis and disease staging. In the context of monoclonal antibody therapy, FDG-PET can be used to monitor treatment-induced changes in brain metabolism, potentially reflecting functional stabilization or recovery despite ongoing structural changes. However, its utility as a standalone efficacy biomarker is limited by relatively low specificity for AD and sensitivity to various confounding factors, including vascular pathology and medication effects. Integrating FDG-PET with structural and molecular imaging may enhance the comprehensive assessment of treatment impact on both pathology and brain function.

### 3.4 Advances in AI-assisted diagnostic technologies

Artificial intelligence (AI) has significantly advanced AD neuroimaging diagnostics. Deep learning algorithms automate multimodal image analysis, enhancing early diagnostic accuracy and efficiency (33). AI models integrate multidimensional data (sMRI, fMRI, PET) to identify subtle pathological patterns, enabling precise disease prediction. In therapy monitoring, AI-assisted software automates detection of treatment-related changes such as ARIA, improving consistency and efficiency. Machine learning algorithms also predict individual responses to monoclonal antibodies based on baseline imaging features, supporting personalized treatment decisions. These advancements are driving AD diagnostics toward precision medicine.

## 4 Multimodal neuroimaging in monoclonal antibody therapy assessment

The comprehensive assessment of monoclonal antibody (mAb) therapy in Alzheimer's disease necessitates a multimodal neuroimaging approach, as no single modality provides a complete picture of treatment effects. Structural, functional, and molecular imaging techniques offer complementary insights, ranging from monitoring safety and quantifying target engagement to evaluating functional neurological outcomes. This integrative strategy is paramount for optimizing treatment protocols, mitigating risks, and advancing personalized medicine. To support clinical decision-making, Table 1 provides a structured framework summarizing baseline imaging requirements, recommended monitoring intervals, key triggers for dose interruption or modification, and guidance on interpreting imaging findings in relation to clinical outcomes for each neuroimaging modality in the context of mAb therapy.

**Table 1:** Imaging-guided decision-making in monoclonal antibody therapy for Alzheimer's disease.

Modality	Baseline requirements (pre-treatment)	Monitoring intervals	Key decision-making triggers	Interpretation relative to clinical outcomes
Structural MRI (sMRI)	Exclude pre-existing ARIA, assess hippocampal volume, cortical thickness, and white matter disease	Every 3 months for first 6 months, then every 6 months; immediate if neurologic symptoms	ARIA-E (edema): mild asymptomatic → continue with close follow-up; moderate-severe → interrupt treatment, consider corticosteroids. ARIA-H (microhemorrhages): new or increasing burden → reassess risk-benefit, consider dose adjustment or discontinuation	Short-term hippocampal volume loss may reflect pseudo-atrophy (amyloid clearance) rather than true neurodegeneration; should not be interpreted as treatment failure without clinical correlation
Aβ-PET	Quantify baseline amyloid burden (centiloid or SUVR) to confirm eligibility and establish reference	Baseline and at 12–18 months	Amyloid reduction threshold: centiloid <25 or SUVR reduction ≥ 50% from baseline → consider extended dosing intervals; minimal reduction → reassess adherence or consider alternative therapy	Robust amyloid clearance correlates with slowing of clinical decline in clinical trials, but individual cognitive response varies; tau burden and baseline cognitive reserve modify the relationship
Tau-PET	Optional: assess baseline tau burden for prognostic stratification	Not routinely repeated; may be considered at 18–24 months if cognitive decline persists despite amyloid clearance	High baseline tau burden predicts faster cognitive decline despite amyloid clearance; may guide intensity of monitoring or consideration of combination strategies	Tau-PET is a stronger predictor of cognitive decline than Aβ-PET; discordant amyloid clearance with persistent tau accumulation may explain suboptimal cognitive response
fMRI	Baseline resting-state connectivity (default mode network) for reference	Baseline and at 18 months; optional mid-treatment if functional changes are of interest	Significant improvement in DMN connectivity may support continued treatment; stable or declining connectivity may prompt investigation of coexisting pathologies	Evidence for fMRI as a treatment response marker remains preliminary; changes should be interpreted as exploratory rather than definitive indicators of efficacy
AI-Assisted Analysis	Integration of multimodal baseline imaging (sMRI, PET, fMRI) for risk prediction	Continuous integration of longitudinal data	Prediction of ARIA risk, treatment response trajectories, and amyloid clearance rates to inform patient selection and adaptive dosing	AI models show preliminary potential but require prospective validation before clinical implementation

### 4.1 sMRI for monitoring cerebral atrophy and treatment response

sMRI provides critical insights into cerebral atrophy progression and treatment response in AD. It accurately measures structural metrics (e.g., hippocampal volume, cortical thickness) strongly correlated with cognitive decline (34, 35). However, the interpretation of sMRI-derived metrics as biomarkers of treatment efficacy is complicated by paradoxical observations of accelerated hippocampal volume loss and cortical thinning in some patients receiving anti-Aβ monoclonal antibodies. This phenomenon, reported in several clinical trials, challenges the straightforward association between structural preservation and therapeutic benefit. Potential mechanisms underlying this apparent acceleration of atrophy include: (1) pseudo-atrophy resulting from the clearance of amyloid plaques and associated edema, leading to a transient reduction in brain volume without true neuronal loss; (2) treatment-related inflammatory responses (e.g., ARIA-E) that may exacerbate local tissue injury and volumetric decline (13, 14); and (3) the possibility of genuine accelerated neurodegeneration in susceptible brain regions due to altered amyloid homeostasis or off-target effects. Importantly, these structural changes may not correlate with clinical worsening and, in some cases, may even precede clinical stabilization or improvement. Therefore, while sMRI remains essential for monitoring safety (e.g., ARIA) and tracking long-term atrophy trends, caution is warranted when interpreting short-term volumetric reductions as indicative of treatment failure or accelerated disease progression (32). Future studies should integrate longitudinal sMRI with complementary biomarkers (e.g., tau-PET, neurofilament light chain) to disentangle therapeutic effects from confounding structural changes and to better define the role of structural imaging in evaluating disease-modifying therapies.

### 4.2 fMRI for evaluating post-treatment functional improvements

fMRI may offer insights into functional connectivity changes following monoclonal antibody therapy, though current evidence for its use as a treatment response biomarker remains preliminary. Resting-state fMRI has been shown to detect alterations in key networks (e.g., default mode network), often preceding structural changes (36). Multilevel functional connectivity analysis combined with graph convolutional networks and random forest algorithms has been applied to predict amyloid-PET patterns, with reported accuracy in Aβ-PET classification (37). Cross-site validation studies suggest that integrated fMRI-sMRI models may achieve superior performance compared to unimodal approaches, indicating potential synergistic value in combining functional connectivity profiles with structural anatomy. However, it should be noted that robust, reproducible evidence for fMRI-based treatment response markers in anti-amyloid monoclonal antibody trials is currently limited, and further prospective studies are needed to validate these preliminary findings.

### 4.3 PET imaging for assessing Aβ clearance

PET imaging remains the gold standard for evaluating Aβ clearance following monoclonal antibody therapy. Aβ-PET directly visualizes changes in amyloid plaque distribution and burden. Clinical studies confirm that anti-Aβ antibodies significantly reduce PET-quantified amyloid load, providing direct evidence of target engagement. A more robust predictor of cognitive decline is tau-PET, which outperforms both Aβ-PET and sMRI, explaining 25% of variance in Aβ-positive mild cognitive impairment cohorts (38). However, high costs and radiation exposure limit its widespread use (39).

### 4.4 AI in predicting therapeutic efficacy

AI technologies show great promise in analyzing multimodal imaging data. Deep learning algorithms integrate sMRI, fMRI, and PET to improve treatment outcome predictions (40). Integrated frameworks combining graph convolutional networks and random forests successfully predict amyloid-PET patterns using fMRI-derived functional connectivity networks. Recent advances have realized techniques that predict individual task-fMRI maps from sMRI data alone have been realized, offering novel avenues for personalized monitoring. AI-assisted systems demonstrate robust generalizability in multicentre validations, providing reliable support for clinical decision-making.

## 5 Therapeutic strategies: targeted interventions and combination therapies

### 5.1 Clinical efficacy of monoclonal antibody therapies

Two anti-Aβ monoclonal antibodies, lecanemab and aducanumab, have received FDA approval for AD treatment, representing the first disease-modifying therapies capable of slowing clinical decline by targeting core biological processes. Clinical trials show lecanemab slows disease progression over 18 months, with PET confirming reduced Aβ burden (41); however, antibodies targeting different Aβ forms yield divergent outcomes, as solanezumab (targeting monomeric Aβ) failed in Phase III trials involving preclinical AD patients (42). A major safety consideration is that these therapies commonly cause adverse events including ARIA, with ARIA-E/H (edema/hemorrhage) being the most frequent radiographic findings (43). To guide safe and effective implementation, a structured imaging workflow is essential. At baseline, structural MRI (sMRI) should exclude pre-existing ARIA and establish a reference for cerebral atrophy, while Aβ-PET quantifies baseline amyloid burden (e.g., centiloid scale or SUVR) to support patient selection. During treatment, sMRI is recommended at 3-month intervals for the first 6 months and every 6 months thereafter, with ARIA-E and ARIA-H graded using standardized scales (13); mild asymptomatic ARIA-E often permits continued treatment with close follow-up, whereas moderate-severe ARIA-E typically requires interruption. For efficacy assessment, Aβ-PET at 12–18 months confirms target engagement through amyloid clearance, and resting-state fMRI at baseline and 18 months may be considered to explore changes in default mode network connectivity and functional reorganization, though its role as a reliable treatment response marker requires further validation.

### 5.2 Multimodal imaging-guided personalized therapy

Multimodal neuroimaging is pivotal in personalizing monoclonal antibody therapies. Brain MRI is essential for monitoring ARIA, with sMRI detecting atrophy and treatment response (44), and fMRI evaluating functional connectivity changes (45); PET imaging directly assesses Aβ deposition, informing therapeutic decisions, while combined amyloid-PET and tau-PET evaluations improve patient selection (32). However, several interpretation pitfalls must be recognized. First, patients receiving anti-Aβ antibodies may show accelerated hippocampal volume loss or cortical thinning during the first 6–12 months despite clinical stabilization, a phenomenon termed “pseudo-atrophy”, which likely reflects clearance of amyloid plaques and reduction of associated edema rather than true neuronal loss (32); clinicians should avoid interpreting such short-term volume reductions as treatment failure. Second, on MRI, ARIA-E appears as hyperintensity on T2-FLAIR sequences and usually resolves spontaneously or with corticosteroids over 4–12 weeks, whereas ARIA-H is best visualized on susceptibility-weighted imaging (SWI) as punctate hypointensities that may accumulate over time (14). Third, significant amyloid clearance on Aβ-PET may occur concurrently with progressive atrophy or ARIA on MRI, reflecting distinct biological processes and underscoring the complementary value of multimodal imaging. Integration of imaging findings with APOE ε4 status enables personalized decision-making: APOE ε4 homozygotes have the highest ARIA risk and may benefit from more frequent MRI surveillance and lower initial doses (14), while patients achieving robust amyloid clearance (e.g., centiloid <25 at 12 months) may be candidates for extended dosing intervals (5). Advances in blood biomarkers may further streamline diagnostics and broaden patient screening, and AI models trained on multimodal baseline imaging (sMRI, fMRI, PET) have shown preliminary potential in predicting individual ARIA risk, treatment response trajectories, and amyloid clearance rates, suggesting a future role in supporting patient selection and adaptive dosing strategies (40); however, prospective validation is needed before clinical implementation.

### 5.3 Imaging assessment of combination therapies

Current research explores combining monoclonal antibodies with other modalities. Dual targeting of Aβ and tau immunotherapy represents a promising direction, while non-pharmacological interventions (e.g., cognitive training, neuromodulation) combined with antibodies are gaining traction. In these complex scenarios, multimodal imaging plays an even more critical role: Aβ-PET and tau-PET can separately quantify the contribution of each therapeutic target, allowing dissection of synergistic effects (32); sMRI monitors structural integrity and ARIA, which may be altered by combined regimens (44); and fMRI captures functional reorganization, particularly relevant for interventions aimed at cognitive rehabilitation (45). AI-assisted multimodal integration enables synthesis of heterogeneous imaging, clinical, and biomarker data to refine predictive models and guide adaptive treatment selection (40). However, combination therapies face challenges including increased costs (46) and complex monitoring requirements, necessitating careful resource planning and prospective validation of standardized imaging protocols.

## 6 Limitations, controversies, and unresolved challenges

### 6.1 Sensitivity and specificity of imaging techniques

Multimodal neuroimaging faces significant sensitivity and specificity limitations in monitoring antibody therapies. The detection of subtle ARIA changes on MRI is highly dependent on multiple factors, including the use of appropriate sequences (FLAIR for ARIA-E, susceptibility-weighted imaging or gradient-recalled echo for ARIA-H), magnetic field strength, reader expertise, and adherence to standardized imaging protocols; suboptimal protocols may lead to underdetection of microhemorrhages or mild edema. PET limitations are two-fold: spatial resolution constraints limit precise anatomical localization of amyloid deposits, and current tracers cannot reliably distinguish soluble Aβ oligomers from fibrillar plaques, which may have different pathophysiological implications (47). Inconsistencies across modalities also arise—e.g., PET may show Aβ clearance while sMRI indicates ongoing atrophy. These limitations contribute to diagnostic uncertainty, underscoring the need for higher-precision biomarkers (48).

### 6.2 Heterogeneous treatment responses to antibodies

Anti-Aβ antibodies exhibit marked interindividual variability. The overall incidence of ARIA varies considerably depending on the specific antibody, dose, and APOE ε4 carrier status, with reported rates ranging from approximately 20% to 40% across different trials. Lecanemab and donanemab show differing ARIA profiles, reflecting antibody-specific safety characteristics and trial populations (5, 14). Approximately 20% of patients show significant Aβ-PET improvement without cognitive benefits, potentially due to unassessed factors like tau burden or neuroinflammation (49). Reliable predictive biomarkers are lacking, hindering individualized risk-benefit assessments.

### 6.3 Long-term efficacy and safety monitoring

Long-term imaging data beyond 18–24 months remain scarce. It is unclear whether sustained Aβ clearance may accelerate tau pathology. ARIA typically occurs early, but long-term consequences (e.g., cumulative microhemorrhages) require larger longitudinal studie. Standardization of MRI sequences and PET quantification across centers is urgently needed to improve data comparability (50). Unified long-term imaging protocols represent a critical future need (51).

## 7 Future research directions and technical pathways

### 7.1 Novel imaging biomarkers

Future studies must prioritize novel biomarkers for earlier and more accurate AD detection. Integrating multimodal imaging phenotypes with genetic data through imaging genomics offers non-invasive insights into AD mechanisms. Techniques like arterial spin labeling MRI (ASL MRI) are being incorporated into the Alzheimer's Disease Neuroimaging Initiative (ADNI) to quantify cerebral blood flow and arterial transit time (52). Combining modalities addresses previously intractable challenges, such as elucidating amyloid neurotoxicity mechanisms.

### 7.2 Integrated analysis of multimodal data

Effective integration of multimodal data is essential. Key challenges include inherent variability across modalities and achieving reliable, interpretable fusion diagnostics (53). Multimachine learning frameworks integrating MRI, PET, and CSF data have improved AD characterization. ADNI's open-data paradigm supports global collaboration through data exploration and coupling techniques (54). Advanced algorithms are needed to handle incomplete multimodal datasets and standardize analytical workflows.

### 7.3 AI in therapeutic decision-making

AI holds immense potential for treatment personalization. Deep learning models integrate multidimensional data to improve diagnostic accuracy and overcome deployment barriers (55). MRI-based deep learning frameworks enhance diagnostic performance through feature fusion (56). Multimodal computational frameworks integrate data from independent cohorts, mitigating reliance on expensive PET imaging (57). AI tools like the Florey Dementia Index (FDI) show promise in predicting MCI-to-AD conversion (58). Future work should prioritize explainable AI models integrated into clinical decision support systems for personalized antibody therapy.

## 8 Conclusions

Multimodal neuroimaging techniques have demonstrated indispensable value in the monitoring and evaluation of monoclonal antibody therapies for Alzheimer's disease. Structural MRI (sMRI) plays a critical role in detecting therapy-related neuroanatomical changes, particularly in the early identification of amyloid-related imaging abnormalities (ARIA) (59). Positron emission tomography (PET) enables direct assessment of cerebral Aβ plaque clearance following monoclonal antibody interventions, providing objective evidence of target engagement. Functional MRI (fMRI) offers dynamic evaluation of treatment-induced improvements in brain network connectivity. Critically, the integration of multimodal imaging enhances diagnostic accuracy and, importantly, provides a comprehensive perspective for evaluating interindividual variability in treatment response. The incorporation of artificial intelligence technologies further augments the depth and efficiency of imaging data analysis, supporting personalized therapeutic decision-making.

Based on current evidence, we propose the following recommendations for clinical practice: First, standardized multimodal imaging protocols should be established, incorporating regular sMRI examinations during monoclonal antibody treatment to monitor ARIA. Second, PET imaging should be integrated into therapeutic response assessments, with Aβ-PET recommended at baseline and mid-treatment to evaluate target clearance. Third, predictive models incorporating multimodal imaging data should be developed, leveraging artificial intelligence to synthesize structural, functional, and metabolic information for improved treatment response forecasting. Fourth, multidisciplinary collaboration should be strengthened through the formation of integrated teams involving neurologists, neuroradiologists, and data scientists to optimize clinical decision-making. Fifth, additional longitudinal studies are needed to explore the relationship between multimodal imaging biomarkers and long-term clinical outcomes, thereby informing personalized treatment strategies. Implementation of these recommendations will help maximize clinical benefits while mitigating treatment-related risks in monoclonal antibody therapy for Alzheimer's disease.
